# Perception and Challenges of Preventive Measures of COVID-19 Among Nepalese Frontline Health Professionals: An Unexplored Realism

**DOI:** 10.3389/fpubh.2021.747070

**Published:** 2022-01-20

**Authors:** Krishna Prasad Pathak, Sandip Das Sanyam, Tara Gaire, Pushpa Babu Basnet, Sanjay Kumar Sah, Buddha Bahadur Basnet, Sujana Pathak, Stan Ingman, Jeremy J. Hoffman

**Affiliations:** ^1^Department of Social Sciences, Nepal Open University, Lalitpur, Nepal; ^2^Center for the Study of Aging, Department of Preventive Medicine, Universidade Federal de São Paulo, São Paulo, Brazil; ^3^Sagarmatha Choudhary Eye Hospital, Lahan, Nepal; ^4^Innovative College of Health Science, Kathmandu, Nepal; ^5^Biratnagar Eye Hospital, Biratnagar, Nepal; ^6^Nepal Academy of Science and Technology, Patan, Nepal; ^7^National Academy of Medical Sciences (NAMS) Ophthalmic, Bir Hospital, Kathmandu, Nepal; ^8^University of North Texas, Denton, TX, United States; ^9^International Centre for Eye Health, London School of Hygiene and Tropical Medicine, London, United Kingdom

**Keywords:** COVID-19, health care personnel (HCP), personal protective equipment (PPE), myths, challenge

## Abstract

**Background:**

A new coronavirus causes COVID-19, a developing respiratory illness. Unfortunately, there is little information assessing healthcare workers' understanding of technology and preventative strategies during the Nepalese epidemic. Researchers from other subspecialties uncovered some mythical thoughts. As a result, we decided to put it to the test with healthcare personnel on the front lines. The research also looked at the problems experienced by frontline health care personnel (HCP) because of the COVID-19 strategic shift in work policy.

**Methods:**

Nepalese healthcare workers participated in web-based cross-sectional research. A pre-tested, structured questionnaire utilizing a Google form was used to get self-informed, digitally typed consent, and examine critical perspectives and problems with current technology and COVID-19 prevention efforts.

**Results:**

In total, 243 participants with mean age of 29.66 ± 7.61 years agreed to participate and were doctors (*n* = 27), health assistants (*n* = 2), medical intern doctors (*n* = 1), paramedical (*n* = 139), pharmacy (*n* = 1), and paramedical interns (*n* = 73) in this study. The calculated mean percentage score of knowledge on instruments and tools was 73.64 (SD ± 10.43) %, and perception on COVID-19 transmission and control was 70.06 (SD ± 18.30) %. At various levels, frontline health workers faced significant challenges, including the adoption of digital health technology.

**Conclusion:**

Frontline HCPs are anticipated to have updated knowledge from what the study has outlined. It is recommended to follow national guidelines. Policies should be put in place so that every frontline worker can demonstrate high standards in prevention, control, and equipment use that do not create misinformation among HCPs. Throughout, support for digital health materials and disease control methods for HCPs is essential.

## Introduction

COVID-19, a coronavirus, is a pandemic globally. There seems to be no other way for the virus to stop its spread other than to prevent it. Proper use of personal protective equipment (PPE) is the key to stopping the spread of the virus from the patient to the caring personnel. The Centers for Disease Control and Prevention (CDC) has given health care personnel (HCP) guidelines for working at health centers and isolation centers (1). Vaccines for the virus have been in the world news but did not reach Nepal until the last day of data collection. Vaccines create an antibody against the virus, but it's not guaranteed that a vaccinated person cannot get infected by the virus, provided the severity is less than that of a diseased non-vaccinated person. So, HCP involved in the frontline must use PPE properly even after the vaccination (2). The latest data have indicated an upsurge in the number of cases in several countries (United Kingdom, India, and Australia) as the second wave of COVID-19 (3–5). South Asian countries' health policies, including Nepal's, appear to be less prepared to deal with such pandemics (6, 7). Thus, to limit the spread of the coronavirus, almost all governments worldwide have “lockdown” as one of their guidelines (8). Furthermore, the Nepal government through the Ministry of Health and Population (MOHP) has been actively broadcasting public awareness messages regarding the prevention and control of the virus through loudspeaker recordings, radio, television, social media, press briefings, etc. Despite all these efforts, there have been reports of the deaths of HCPs throughout the world, and Nepal was not spared either (9–11). This could be due to many reasons, like inattention to the appropriate use of PPE in situations of anxiety, depression, traumatic stress, and burnout due to COVID-19 (12, 13), or just a simple myth, mask-carbon dioxide intoxication in prolonging usage (14), can wear one mask for several days with/without washing, belief in inappropriate use of mask (15); gloves can mimic the spread of the virus (16); thermal scanner: all COVID-19 infections can be detected as a result of high temperatures (14); polymerase chain reaction (PCR) dilemma: it is not superior, and it gives favorable/adverse effects just like that, even if we are scared/asymptomatic, short of misleading (17). So, logically, HCP could become infected with COVID-19 regardless of preventive measures taken, because they are the ones who work on the frontlines of Nepal's and the rest of the world's communities (18). It is also possible that one could get infected just by chance with an unknown etiology, irrespective of any conditions. Research is still evolving in studying the broad etiology of this coronavirus.

Digital technologies and protective instruments used in testing, treatment, and management of COVID-19 are the pillars for protecting and restricting the spread of diseases among people, especially for the frontline HCPs (19, 20). Although the advancement of digital technology has a significant positive impact in situations such as COVID-19, there appear to be several challenges in implementing it in a suitable manner (21, 22).

A pilot test of the same tool revealed poor knowledge of the HCP. Hence, this study was conducted to access the knowledge of current critical technology and understand the perceptions of health professionals toward the spread and control of the pandemic. In addition, we looked at the challenges faced in treating the patients and adapting the digital health material to prevent COVID-19 among frontline HCP in Nepal with the help of a comprehensive yet straightforward questionnaire tool.

## Methodology

### Materials

A web-based qualitative descriptive cross-sectional study was carried out from August 10 to December 23, 2020, among frontline healthcare workers. The Nepal Health Research Council (NHRC) granted permission to conduct the study with mandatory written consent as the criterion. Each participant was asked to sign a digitally written and informed consent form. The research tool and design were adopted and streamlined from Sanyam's et al. (23) work. Unlike the previous study with multiple questions, we shortened the previous tool and created three simple ways to analyze the critical views of frontline health workers working during the COVID-19 pandemic: (1) perception of COVID-19 virus transmission and control; (2) perception of instruments and tools; and (3) challenges. Each perception heading consisted of five questions, whereas the challenges heading had two questions, all to be answered mandatorily. All the questions had one-word answers. The questionnaire tool can be viewed in the Supplementary File as an annexure questionnaire tool.

### Method

A qualitative method was implemented to construct the questionnaire of perceptions and challenges. It is demonstrated that questions created this way enhance the quality and emphasize the validity of the subject matter (24). The final questions elicited by this technique are tailored to the target population rather than researchers. Furthermore, focus group discussion among public health researchers, statisticians, and professors supported the reliability of the test and retest method, which were used twice in a 10-day interval on the same pilot population. This survey was expected to take no more than 10 min to complete for the respondents as it was voluntary and anonymous. The survey invitations were sent to 742 (altogether 82 COVID-19 dedicated centers are there in Nepal as per MOHP, and if we assume an average of 40 COVID-19 dedicated frontline health workers at each center, then it is about 22% of the frontline health workers) participants working in the COVID-19 centers of all seven provinces of Nepal through What's App, Facebook Messenger, Viber, Telegram, and email. One person (known through personal contact) at each center was given the responsibility to circulate it to other frontline workers in their community and inform the primary investigator about their social media account if a reminder message was needed. The study population includes medical and paramedical health personnel working on the frontline at COVID-19 centers. After their acceptance, the survey took them to the questionnaire section. Participants were allowed to withdraw their participation any time before the first week of February 2021 without experiencing any disadvantage. The same person was not allowed to take the survey twice. This was countered with the help of unique email identification bracketing from the Google form, and those who didn't respond the first time were sent a reminder twice in 2 weeks. The information's confidentiality was guaranteed through protecting privacy.

The “Yes” option added one point to the respective question, whereas the “No” option also gave one point, provided a respondent could select only one option for each question. The correct answers were averaged based on the perception group mentioned above. The perception of disease transmission and instruments were analyzed as the calculated mean percentage of overall responses. The obtained percentage was further graded as “Excellent” if it was >80%, “Satisfactory” if it was >65%, and “Poor” if it was <55% as a total. The options-based response of an individual was used to analyze challenges.

### Data Analysis

The response record was exported from Google form to Microsoft Excel 365 to clean the data, and later, the Statistical Package for Social Sciences (version 20.0) for statistical analysis was used. Finally, the data were presented in percentage, mean, standard deviations (SD), and frequency using bar diagrams and tables.

## Results

The survey questionnaire was sent to 742 people, of whom 243 (male: 118, female: 125), mean age of 29.66 (SD ± 7.61) years, agreed to participate, whereas three did not consent from the list of overall respondents. Participants were doctors (*n* = 27), health assistants (HA, *n* = 2), medical intern doctors (*n* = 1), paramedical (*n* = 139), pharmacy (*n* = 1), and paramedical interns (*n* = 73) in this study. The remaining 496 did not turn up even after the second reminder. The calculated response rate was 32.74%.

The details of socio-demographics are presented in Table 1. The majority of participants were from province three and the least from province six. Most health professionals have a bachelor's degree regarding educational background, except for HAs and proficiency nurses. All of them, with and without prior work experience, work as frontliners. In terms of work setting, most frontline health professionals were employed in health institutions as full-timers with a continuous 8-h duty base. In addition, all health care professionals were involved in direct care like swab collection, fever reporting, long-term (admitted patients' care) and short-term (OPD clinics), laboratory department, dead body management, x-ray department, nursing care, pharmacy, etc. using masks, round caps, gloves, protective gowns, boot covers, and goggles or face shields.

**Table 1:** Socio-Demographic characteristics.

**Variables**		**Frequency**	**Percentage**
Gender	Male	118	48.6
	Female	125	51.4
Province	Province 1	66	27.2
	Province 2	28	11.5
	Province 3	91	37.4
	Province 4	14	5.8
	Province 5	40	16.5
	Province 6	1	0.4
	Province 7	3	1.2
Types of health person	Doctor	27	11.1
	HA	2	0.8
	Medical intern	1	0.4
	Paramedical	139	57.3
	Pharmacy	1	0.4
	Paramedical intern	73	30

There is a scarcity of protective equipment, exacerbated in low- and middle-income countries due to limited resources. Health workers were self-motivated to know and be updated about this pandemic using government websites, awareness messages, and social media. That means a well-associated positive attitude toward obtaining the information. In this study, we asked about the instruments, the virus transmission, and control. About 50% (*n* = 127) felt that PCR is mandatory for everyone, whereas a similar (*n* = 116) number of responses were noted for temperature screening, which was not helpful in screening for COVID-19. A limited yet considerable (about 10%, *n* = 19) number of participants were unsure about the importance of teleconsultation in contagious diseases like COVID-19. The obtained detailed perception responses are tabulated in Table 2.

**Table 2:** Perception on COVID-19 transmission and control.

**Knowledge**	**Yes *n* (%)**	**No *n* (%)**
Everyone must get a PCR test done	**127 (52.3)**	116 (47.7)
Hygiene training is necessary for the spread of viruses	**206 (84.8)**	37 (15.2)
Fever screening helped to prevent the spread of viruses	**167 (68.7)**	76 (31.3)
Temperature screening is helpful to screen COVID-19 infected patients	**127 (52.3)**	116 (47.7)
Teleconsultations reduce the risk of disease spread	**224 (92.2)**	19 (7.8)

*The actual answers are in bold and non-bold indicates the incorrect responses from the participants*.

The calculated mean knowledge score of perception on COVID-19 transmission and control was 70.06% (SD ± 18.30), at a satisfactory level.

**Figure 1:**
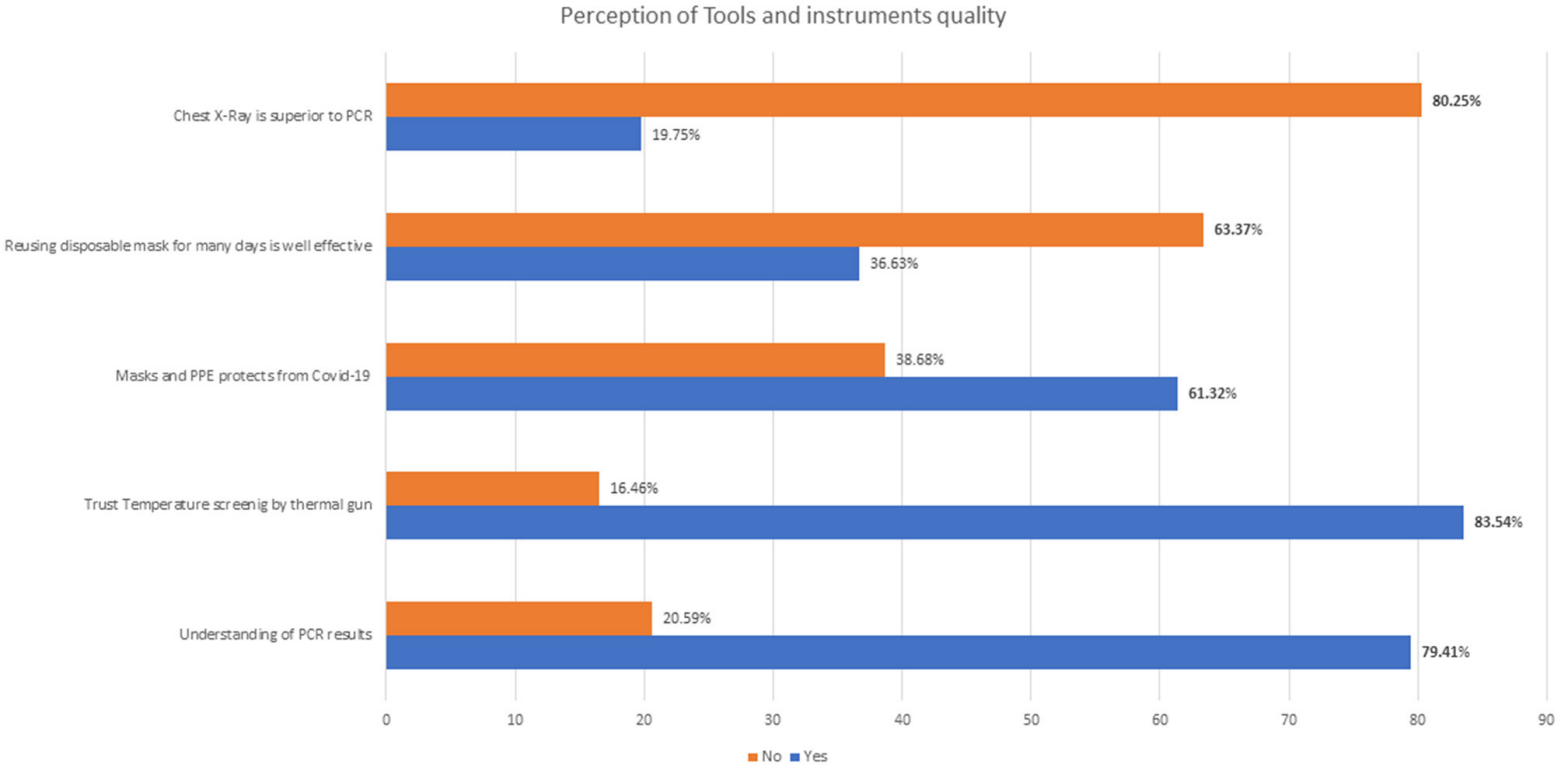
Responses regarding perception on instruments and tools. Bold percentage values indicate the true/expected answers.

Figure 1 shows the descriptive analysis of responses related to perceptions of instruments and tools. Participants gave their agreement (80.25%) to Polymerase Chain Reaction (PCR) superiority over chest X-ray. Similarly, they responded positively to the masks not being effective after many days of use, but surprisingly, they still don't (38.68%) believe that masks and PPE are 100% effective. About 21% of those polled had no understanding of how to interpret PCR data.

The calculated mean percentage score of perception on instruments and tools was 73.64% (SD ± 10.43), at a satisfactory level.

## Challenges

There was a significant issue with adapting and using the protective measures during the pandemic, and this had a drastic impact on the lifestyle and working habits of the health professionals. In Table 3, we have illustrated the challenges of coping with the restrictions and adjustments to the lifestyle in the day-to-day COVID-19 wave. Very few (1.6%, *n* = 4) who did not encounter any problems adapting to COVID-19 made variations in health strategies. In contrast, the majority (72.4%, *n* = 176) agreed on challenges faced at personal, institutional, technological, and policy levels.

**Table 3:** Challenges faced by health professionals.

**Challenges**		**Frequency**	**Percentage**
During the COVID-19 pandemic	Personal	20	8.3
	Institutional	20	8.3
	Technological	11	4.5
	Policy	12	4.9
	All the above	176	72.4
	None	4	1.6

**Figure 2:**
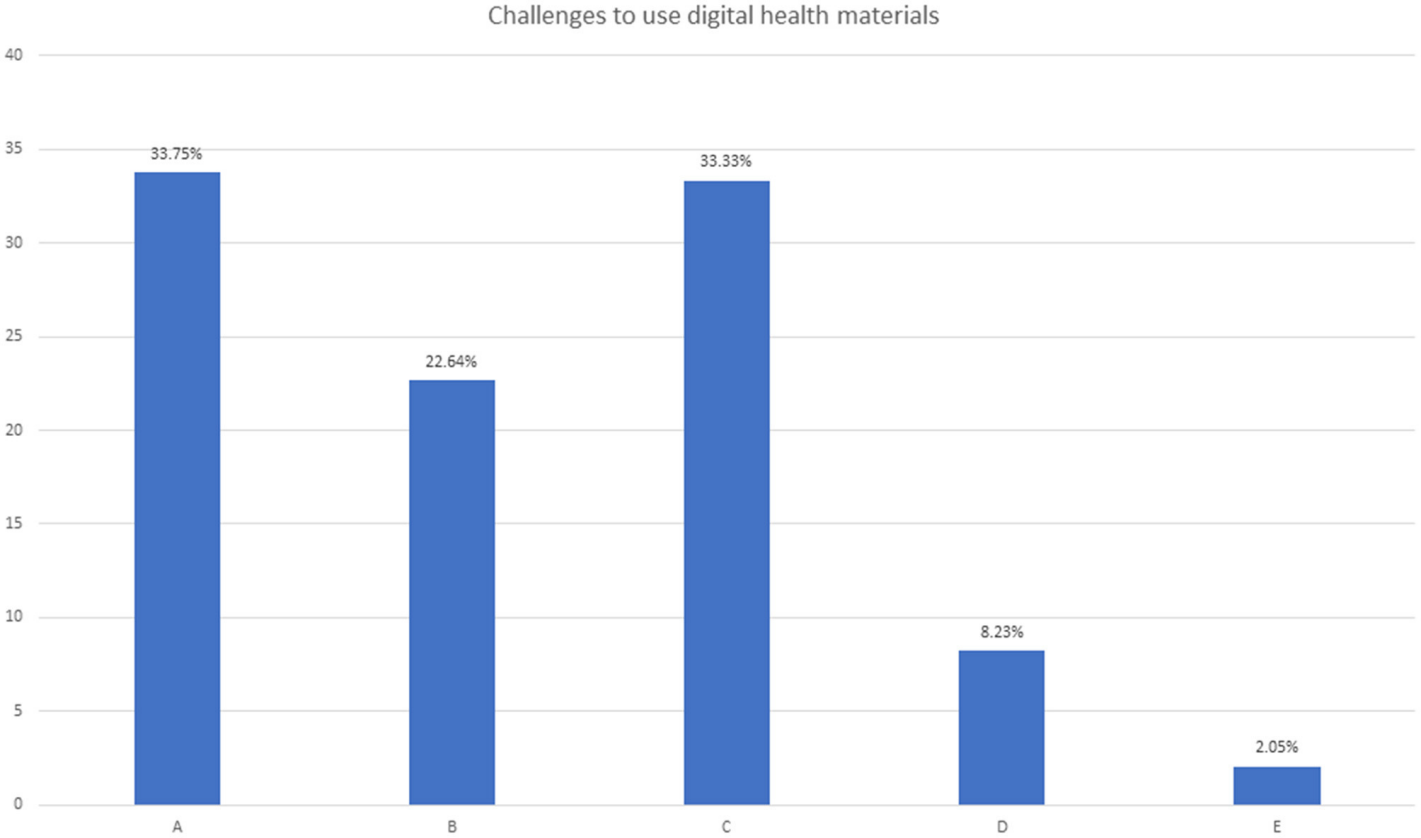
Responses regarding challenges to using digital health materials. In the figure, A = Lacking proper training, B = Lacking the monitoring and supervision, C = Lacking the technical support, D = None, E = all of the above.

Similarly, the challenges were in using digital health materials. Health systems have started to emphasize telemedicine and teleconsultation for non-emergency cases. Institutions have moved their educational delivery and examination system to digital platforms. The below bar diagram shows that the highest (33.75%, *n* = 82) challenge faced was lack of appropriate training to use digital health and educational materials. Only 8.2% (*n* = 20) of health professionals did not have any sort of difficulties in adapting to and using digital devices to make the health system more effective. The overall challenge of using digital health materials by frontline health workers during COVID-19 is shown in the bar diagram (Figure 2).

## Discussion

Even the well-established health systems in the world are hit very hard by COVID-19, leading to a public health crisis (25). Despite all the public health problems, HCPs accepted it as the “new normal” and kept their service continuing to serve humankind using PPE and other protective measures. The perception of frontline HCP on COVID-19 transmission and control, instruments, and tools was satisfactory. There were challenges in dealing with the COVID-19 pandemic at different levels and the challenge of using digital health materials. The knowledge of disease transmission, control, and instruments necessary for the frontline HCPs is sensitive, and HCPs must reflect it with an updated and clear understanding.

Altogether, 69.33% of the participants felt that awareness messages and hygiene training regarding COVID-19 by MOHP were essential in controlling the spread of the virus. PPE is considered a shield for virus transmission from the patient to the frontline HCP (26). But, the uniform sizing for all and the long hours of wearing might cause discomfort, leading to body parts of HCPs working at critical care units (27). Visual impairment makes it difficult to communicate quickly, especially for older inpatients with impaired hearing, as noted by Hampton et al. (28), while there is no doubt about the positive effects of PPE. There were barriers to the resulting “hello hi effect,” “intimacy,” and “closeness” between patients and staff. The muffled voices of health professionals and facial expressions using monosyllabic words were not perfectly adequate. It reduced the understanding between the health staff in the operation theater (OT) and the intensive care unit (ICU). Despite this level of stress and the mortality of patients in the ICU, HCPs don't give up and unknowingly might pile up anxiety, depression, and work-related burnout (12). This may result in careless performance and may be a factor in the death of frontline HCPs. About 39% of the participants said that PPEs don't work well. Similarly, 36.97% thought that reusing daily disposable masks was effective. This is a contagious belief that medical/surgical daily disposable masks don't protect against COVID-19 if used for a long time (29). Wearing a mask for longer working hours doesn't cause carbon dioxide intoxication (14), as no study has proved it so far.

There were some valid concerns raised about the dependability and validity of temperature-sensing technologies such as the thermal gun. Our question on thermal gun and temperature screening resulted in 83.61% saying it works well, and 84.87% disagreeing that “Temperature screening is helpful to screen COVID-19 infected patients,” but 52.52% believed that screening fever helps to prevent the spread of COVID-19. Thermometers have been in existence since the 1600s in clinical practice. Still, forehead hitting may affect variety of factors (anti-fever medicine use, sunlight, hormonal production, external environment, physical activities, hot beverages, pregnancy, circulatory issues, stress, etc.). All those factors can impact the body temperature; thus, it may not be adequate to declare the thermal gun report as standard care (30), but it's okay to use it as a screening device.

PCR is considered the gold standard in the diagnosis of COVID-19. However, the understanding of PCR seemed insufficient in this survey, as 20.59% of the participants could not interpret the PCR result and seemed to have poor knowledge. Similarly, 19.33% believed that chest X-ray is superior to PCR in the COVID-19 suspects. However, roughly half of the participants, 52.10%, said that everyone must get a PCR test done with or without symptoms. This is not the fact; the reality is that it is mandatory to have knowledge about PCR and get it done if the patient has symptoms or is asymptomatic but exposed to COVID-19 aerosols (31, 32). Various countries also ask for mandatory adverse COVID-19 PCR reports from travelers across the border. The news about fake reporting and paid negative PCR reports has created cloudiness in the view of HCP in Nepal, leading to a situation of fear and dilemma simultaneously (17, 31, 33). The government should have strong regulatory rules (punishable offense) and an inspection team to bring up the standards and trust in health and laboratory reports.

The challenges faced by HCP are under-studied by the nation, which is a matter of concern. About two-thirds (72.4%, *n* = 176) of the participants said they faced challenges at all levels (personal, institutional, technology, and policy) in adopting the change in health strategy amended due to COVID-19. The government (MOHP) should take this as a matter of seriousness and support the frontline HCPs in a more effective implementation model (34). Telemedicine and digital health service delivery are considered a boon for the health system, specifically in conditions like the COVID-19 pandemic (35). In a country like Nepal, where development has not reached every corner of the country, there are places where no transportation service is available, and one should walk for 2–3 days to get there. The internet, telephone networks, and electricity are not accessible equally around the nation, which is required in digital technologies. The bar graph in Figure 2 gives an idea of the challenges (no or inadequate training, 33.75%; inadequate monitoring and supervision, 22.64%; and poor/no technological support, 33.33%) faced by frontline HCPs. Digital services like telemedicine, smartphone tracking and tracing, virtual training, operating sophisticated instruments, etc., need specialized training, monitoring, and support (36–38). Health professionals lacking the proper training to use digital technologies, supervision, and technical support do not add to the security and confidence they lack, resulting in poor implementation strategy and outcome.

Since this was an online study, the response rate and the number of participants were lower. Although only one-third of the participants responded to the survey, although it was randomly selected, it may have led to selection bias. Because of the need for timely data during a pandemic and a lack of proper resources with nationwide restrictions, a non-probability (convenience) sampling method was chosen. The COVID-19 pandemic is still evolving, and there might be a change in perception throughout the data collection until the publication of the report. Although the sampling technique was non-probability-based, the overall sample of this study is sufficient to draw the required conclusion. This study has the potential to be useful in highlighting some of the public health concerns and implementation issues that are mandatory for the Nepal government to consider. Also, there is critical public interest in understanding health workforce capacity and strain during the COVID pandemic. The government should carry out more robust training and awareness for frontline healthcare workers to recompense any misinformation. Also, it is recommended that MOHP carry out a similar but detailed study at the policy level, making it mandatory for all HCPs to analyze the gap and develop a strategic work plan to overcome the challenges and update knowledge.

## Conclusion

Although the perception of instruments and tools and disease transmission and control scores seemed satisfactory in HCP, it cannot be considered acceptable for public health concerns like COVID-19. Inadequate information about disease control, communication, and tools employed are indicators of poor health outcomes. Some of the perceptions were so based on fiction that they may have implications for hospitals, COVID centers, and respective government bodies. HCPs must seek out relevant information on disease management and continue to upgrade their skills following MOHP regulations. The difficulties encountered by HCPs in this study are expected to obstruct the result. So, the challenges faced by HCP should be addressed with the appropriate strategy regularly. Any form of training given to HCP must have 24/7 support in terms of tech or monitoring.

## Data Availability Statement

The original contributions presented in the study are included in the article/Supplementary Material, further inquiries can be directed to the corresponding author/s.

## Ethics Statement

The studies involving human participants were reviewed and approved by Nepal Health Research Council. The patients/participants provided their written informed consent to participate in this study.

## Author Contributions

SDS, KP, TG, and BB designed the study. KP, SDS, JH, and BB reviewed the design. SDS, SKS, KP, SP, TG, and PB collected and cleaned the data. KP and BB oversaw the study. SDS drafted the manuscript. KP, JH, and SI crucially evaluated the manuscript. SDS, KP, and JH revised the reviewers' comments extensively. All authors analyzed the data and have authorized the final manuscript for publication.

## Conflict of Interest

The authors declare that the research was conducted in the absence of any commercial or financial relationships that could be construed as a potential conflict of interest.

## Publisher's Note

All claims expressed in this article are solely those of the authors and do not necessarily represent those of their affiliated organizations, or those of the publisher, the editors and the reviewers. Any product that may be evaluated in this article, or claim that may be made by its manufacturer, is not guaranteed or endorsed by the publisher.

## References

[1] Centers for Diseases Control Prevention. Interim Infection Prevention and Control Recommendations for Healthcare Personnel During the Coronavirus Disease 2019 (COVID-19) Pandemic. Health Care Workers (2021). Available online at: https://www.cdc.gov/coronavirus/2019-ncov/hcp/infection-control-recommendations.html

[2] Centers for Diseases Control Prevention. Updated Healthcare Infection Prevention and Control Recommendations in Response to COVID-19 Vaccination. Healthcare Workers (2021). Available online at: https://www.cdc.gov/coronavirus/2019-ncov/hcp/infection-control-after-vaccination.html

[3] Coates S. 2nd Wave of Coronavirus in Countries Around Asia Prompts Fresh Lockdowns. Global News (2020). Available online at: https://globalnews.ca/news/7220628/asia-second-wave-coronavirus-lockdowns/

[4] News B. Covid-19: Prime Minister Says UK 'Seeing a Second Wave'. British Boardcasting Corporation (2020). Available online at: https://www.bbc.com/news/uk-54212654

[5] Mewada P. How Can We Survive The Second Wave Of COVID-19? PharmEasy Blog (2021).

[6] Basnet B Bishwakarma K Pant R Dhakal S Pandey N Gautam D. Combating the COVID-19 pandemic: experiences of the first wave from Nepal. Front Public Health. (2021) 9:613402. 10.3389/fpubh.2021.613402 34322466 PMC8310916

[7] Chalise HN Pathak KP. Situation of COVID-19 pandemic in South Asia. J Health Alli Sci. (2020) 10:11–4. 10.37107/jhas.18433200035

[8] Pathak K Gaire T Acharya D. Novel coronavirus disease (COVID-19): social distancing, isolation and quarantine are key success factors of Nepal's public Health practices or something else? Kathmandu Univ Med J. (2020) 18:68–74. 10.3126/kumj.v18i2.33034 33605242

[9] Pandey N Basnet B Koju S Khapung A Gupta A. Awareness of Aerosol Related Transmission of COVID-19 Among the Dentists of Nepal. (2021). [preprint] 10.21203/rs.3.rs-499197/v1 34183657 PMC8237251

[10] Berger D. Up the line to death: covid-19 has revealed a mortal betrayal of the world's healthcare workers. BMJ Opin. (2021). Available online at: https://blogs.bmj.com/bmj/2021/01/29/up-the-line-to-death-covid-19-has-revealed-a-mortal-betrayal-of-the-worlds-healthcare-workers/

[11] Amnesty International. COVID-19: Health Worker Death Toll Rises to at Least 17000 as Organizations Call for Rapid Vaccine Rollout. Amnesty International (2021). Available online at: https://www.amnesty.org/en/latest/news/2021/03/covid19-health-worker-death-toll-rises-to-at-least-17000-as-organizations-call-for-rapid-vaccine-rollout-2/

[12] Pappa S Athanasiou N Sakkas N Patrinos S Sakka E Barmparessou Z. From recession to depression? Prevalence and correlates of depression, anxiety, traumatic stress and burnout in healthcare workers during the COVID-19 pandemic in Greece: a multi-center, cross-sectional study. Int J Environ Res Public Health. (2021) 18:2390. 10.3390/ijerph18052390 33804505 PMC7967750

[13] Chatterjee SS Bhattacharyya R Bhattacharyya S Gupta S Das S Banerjee BB. Attitude, practice, behavior, and mental health impact of COVID-19 on doctors. Indian J Psychiatry. (2020) 62:257–65. 10.4103/psychiatry.IndianJPsychiatry_333_20 32773868 PMC7368446

[14] World Health Organisation. The Prolonged Use of Medical Masks* When Properly Worn, Does Not Cause CO2 Intoxication Nor Oxygen Deficiency. Mythbusters (2020). Available online at: https://rb.gy/jmu57z

[15] World Health Organisation. Coronavirus Disease (COVID-19) Advice for the Public: When and How to Use Masks. All About Masks in the Context of COVID-19. World Health Organisation (2020). Available online at: https://rb.gy/tuezxn

[16] World Health Organisation. It is Safer to Frequently Clean Your Hands and not Wear Gloves. Mythbusters (2020). Available online at: https://rb.gy/jmu57z

[17] Mandal CK. Fake Negative Test Reports Cause for Concern. Kathmandu: The Kathmandu Post (2021).

[18] Nguyen LH Drew DA Graham MS Joshi AD Guo CG Ma W. Risk of COVID-19 among front-line health-care workers and the general community: a prospective cohort study. Lancet Public Health. (2020) 5:e475–83. 10.1016/S2468-2667(20)30164-X 32745512 PMC7491202

[19] Ting DSW Carin L Dzau V Wong TY. Digital technology and COVID-19. Nat Med. (2020) 26:459–61. 10.1038/s41591-020-0824-5 32284618 PMC7100489

[20] Güner HR Hasanoglu I Aktaş F. COVID-19: prevention and control measures in community. Turk J Med Sci. (2020) 50:571–7. 10.3906/sag-2004-146 32293835 PMC7195988

[21] Feroz AS Khoja A Saleem S. Equipping community health workers with digital tools for pandemic response in LMICs. Arch Public Health. (2021) 79:1. 10.1186/s13690-020-00513-z 33390163 PMC7779158

[22] Pathak KP Gaire T Laya A Neumann APFM Conti MdSB Cohrs FM. Implementations of digital technologies in COVID−19 pandemic and other health threats: multi nationals responses. Res Soc Dev. (2021) 10:e172101421776. 10.33448/rsd-v10i14.21776

[23] Sanyam SD Sah SK Chaudhary P Burton MJ Hoffman JJ. Knowledge and awareness-based survey of COVID-19 within the eye care profession in Nepal: misinformation is hiding the truth. PLoS ONE. (2021) 16:e0254761. 10.1371/journal.pone.0254761 34288939 PMC8294537

[24] Azizi N Karimy M Abedini R Armoon B Montazeri A. Development and validation of the health literacy scale for workers. Int J Occup Environ Med. (2019) 10:30–9. 10.15171/ijoem.2019.1498 30685775 PMC6522212

[25] Bhaskar S Rastogi A Chattu VK Adisesh A Thomas P Alvarado N. Key strategies for clinical management and improvement of healthcare services for cardiovascular disease and diabetes patients in the coronavirus (COVID-19) settings: recommendations from the REPROGRAM consortium. Front Cardiovasc Med. (2020) 7:112. 10.3389/fcvm.2020.00112 32613010 PMC7308556

[26] World Health Organization. Rational Use of Personal Protective Equipment (PPE) for Coronavirus Disease (COVID-19): Interim Guidance, 19 March 2020. World Health Organization (2020). Available online at: https://apps.who.int/iris/handle/10665/331498

[27] Yuan N Yang W-X Lu J-L Lv Z-H. Investigation of adverse reactions in healthcare personnel working in level 3 barrier protection PPE to treat COVID-19. Postgrad Med J. (2021) 97:351–4. 10.1136/postgradmedj-2020-137854 32554543 PMC7301344

[28] Hampton T Crunkhorn R Lowe N Bhat J Hogg E Afifi W. The negative impact of wearing personal protective equipment on communication during coronavirus disease 2019. J Laryngol Otol. (2020) 134:577–81. 10.1017/S0022215120001437 32641175 PMC7387788

[29] Wang D Sun B-C Wang J-X Zhou Y-Y Chen Z-W Fang Y. Can masks be reused after hot water decontamination during the COVID-19 pandemic? Engineering. (2020) 6:1115–21. 10.1016/j.eng.2020.05.016 32837748 PMC7320690

[30] Fletcher T Whittam A Simpson R Machin G. Comparison of non-contact infrared skin thermometers. J Med Eng Technol. (2018) 42:65–71. 10.1080/03091902.2017.1409818 29493342

[31] Regional Medical Laboratory. COVID-19 Test Myths and Clarifications. (2021). Available online at: https://www.rmlonline.com/images/data/attachments/0000/2446/Covid19_Test_Myths_and_Clarifications_1_26_21.pdf

[32] Healthline. When Should You Get a COVID-19 Test? What About an Antibody Test? Healthline News (2020). Available online at: https://rb.gy/kgm6yd

[33] The Himalyan News Service. Fake PCR Reports Made. Bajura: The Himalyan (2020). Available online at: https://thehimalayantimes.com/nepal/fake-pcr-reports-made

[34] Dutta U Sachan A Premkumar M Gupta T Sahoo S Grover S. Multidimensional dynamic healthcare personnel (HCP)-centric model from a low-income and middle-income country to support and protect COVID-19 warriors: a large prospective cohort study. BMJ Open. (2021) 11:e043837. 10.1136/bmjopen-2020-043837 33619195 PMC7902325

[35] Giansanti D Aprile I. Letter to the editor: is the COVID-19 pandemic an opportunity to enlarge the telemedicine boundaries? Telemed e-Health. (2020) 26:1123–5. 10.1089/tmj.2020.0159 32456559

[36] Mather CA Cummings E. Developing and sustaining digital professionalism: a model for assessing readiness of healthcare environments and capability of nurses. BMJ Health Care Inform. (2019) 26:e100062. 10.1136/bmjhci-2019-100062 31676494 PMC7062341

[37] Cooper RB Zmud RW. Information technology implementation research: a technological diffusion approach. Manage Sci. (1990) 36:123–39. 10.1287/mnsc.36.2.123

[38] Wu J-H Wang S-C Lin L-M. Mobile computing acceptance factors in the healthcare industry: a structural equation model. Int J Med Inform. (2007) 76:66–77. 10.1016/j.ijmedinf.2006.06.006 16901749

